# Coagulation and Skin Autoimmunity

**DOI:** 10.3389/fimmu.2019.01407

**Published:** 2019-06-20

**Authors:** Massimo Cugno, Alessandro Borghi, Simone Garcovich, Angelo Valerio Marzano

**Affiliations:** ^1^Dipartimento di Fisiopatologia Medico-Chirurgica e dei Trapianti, Università degli Studi di Milano, Milan, Italy; ^2^Medicina Interna, Fondazione IRCCS Ca' Granda Ospedale Maggiore Policlinico, Milan, Italy; ^3^Sezione di Dermatologia e Malattie Infettive, Dipartimento di Scienze Mediche, Università degli Studi di Ferrara, Ferrara, Italy; ^4^Istituto di Dermatologia, Università Cattolica del Sacro Cuore, Fondazione Policlinico A. Gemelli IRCCS, Rome, Italy; ^5^UOC Dermatologia, Fondazione IRCCS Ca' Granda Ospedale Maggiore Policlinico, Milan, Italy

**Keywords:** coagulation, autoimmunity, urticaria, angioedema, bullous pemphigoid, psoriasis, atopic dermatitis, dermatitis herpetiformis

## Abstract

Several lines of evidence indicate that the immune system, inflammation, and coagulation are simultaneously activated in autoimmune and immune-mediated skin diseases. Pro-inflammatory cytokines such as interleukin-6 and tumor necrosis factor-alpha induce the expression of the main initiator of coagulation, i.e., tissue factor. The proteases of coagulation in turn act on protease-activated receptors inducing the expression of various pro-inflammatory cytokines triggering inflammation. The cross-talk among immune system, inflammation, and coagulation amplifies and maintains the activation of all three pathways. This review focuses on three skin disorders as chronic spontaneous urticaria (CSU), angioedema, and bullous pemphigoid (BP), in which the relationships among the three systems have been investigated or their clinical consequences are relevant. Markers of thrombin generation, fibrinolysis, and inflammation have been reported to be increased in the plasma during flares of CSU and angioedema, as well as in the active phase of BP, with the marker levels reverting to normal during remission. The coagulation activation seems to be important only at local level in CSU and angioedema while both at local and systemic levels in BP which is the only condition associated with an increased thrombotic risk. The prothrombotic state in autoimmune skin diseases raises the question of the indication of anticoagulant treatment, particularly in the presence of other cardiovascular risk factors.

## Introduction

Immune system, blood coagulation and inflammation strictly interact in providing a defense against a variety of potentially injurious stimuli, such as infections and tissue damages (1). The molecular mechanisms of this interaction have been largely elucidated. Indeed, pro-inflammatory cytokines, like interleukin 1 (IL-1), IL-6, and tumor necrosis factor alpha (TNF-α), induce the expression of tissue factor (TF) the main initiator of blood coagulation whereas downregulate the natural anticoagulants such as antithrombin, protein C, and TF pathway inhibitor (2). On the other hand, the coagulant mediators (FVIIa, FXa, and FIIa) in turn act on protease-activated receptors (PAR) inducing the expression of pro-inflammatory cytokines (3, 4). The relationships between the activation or dysfunction of the immune system and the coagulation system are evident in systemic autoimmune or immune-mediated diseases including lupus erythematosus (5, 6), rheumatoid arthritis (7, 8), and inflammatory bowel diseases (9, 10) which show an increased risk of thrombosis. A few studies suggested the involvement of blood coagulation also in some immune-mediated skin disorders whose aspects will be analyzed in the present review.

Immune-mediated inflammatory skin diseases comprise a group of heterogeneous chronic disorders that share similar immune-mediated pathogenic mechanisms as well as genetic susceptibility. Although their specific etiologies remain often unknown, all are recognized to involve dysregulation of the immune system, including an over-expression of the pro-inflammatory cytokines. In autoimmune and immune-mediated skin disorders, the cross-talk between inflammation and coagulation creates a self-refueling loop which amplifies and sustains the activation of both systems (3). Growing evidence suggests that this has both local and systemic implications.

The aim of the present study is to focus on several skin disorders in which the relationships between immune response, inflammation and blood coagulation have been investigated and/or their clinical consequences are relevant such as chronic spontaneous urticaria, angioedema, and bullous pemphigoid.

## Chronic Spontaneous Urticaria

Urticaria is a common skin disease characterized by short-lived swellings, called wheals, which resolve in <24 h. Urticaria can be classified according to duration and etiology as acute or chronic (11). Chronic urticaria is defined as urticaria with or without angioedema lasting more than 6 weeks and can be further classified according to whether it is inducible or not into chronic inducible urticaria or chronic spontaneous urticaria (CSU).

**Figure 1:**
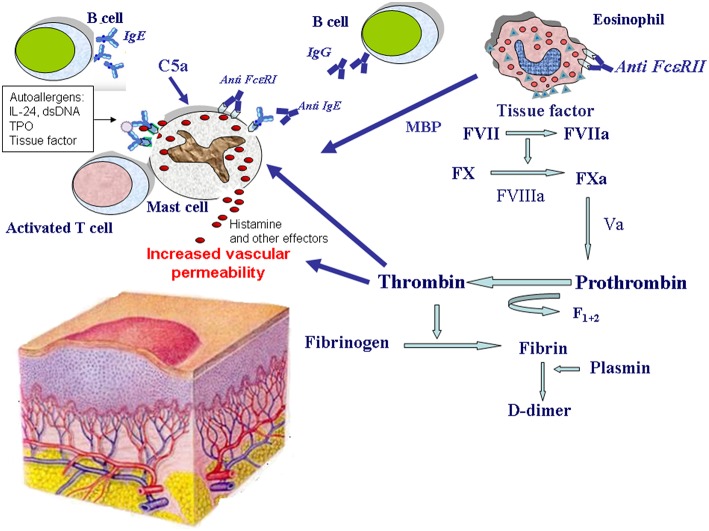
Mechanisms of eosinophil and mast cell activation in chronic spontaneous urticaria. Mast cells release histamine and other effector molecules after stimulation by autoantibodies directed against the high-affinity IgE receptor (FcεRI) and IgE, complement anaphylatoxin C5a, eosinophil-derived major basic protein (MBP), thrombin, and IgE-autoallergen complexes. The most important autoallergens are: interleukin-24 (IL-24), double stranded DNA (dsDNA), thyroperoxidase (TPO), tissue factor. Eosinophils are activated by autoantibodies directed against the low-affinity IgE receptor (FcεRII) and potentially by other factors. Upon their activation, eosinophils release MBP, and express tissue factor which in turn activates the coagulation cascade (factors VII, X, VIII, V, and prothrombin) leading to thrombin generation. Thrombin induces mast cell degranulation as well as increased vascular permeability. Thrombin generation is demonstrated by the presence of fragment F1+2 released from prothrombin after its activation. Finally, fibrin degradation is documented by elevated plasma levels of the fibrin fragment D-dimer during the active phase of the disease.

Despite the great research effort of the last 20 years, etiology and pathogenesis of CSU remain largely unclear. However, there is growing evidence that different biologic systems including autoimmunity (12–14), inflammation (15–18), coagulation (19), and auto-allergy (20–23) are involved in the mechanisms leading to mast cell and basophil degranulation and hence to wheal formation (Figure 1).

The autoimmune mechanism is based on the presence of circulating histamine-releasing IgG autoantibodies directed against either the high-affinity IgE receptor (FcεRI) on both mast cells and basophils (in most cases) or membrane-bound IgE (in a minority of patients) (12, 13, 24). In a variable proportion (30–60%) of patients with active disease, the intradermal injection of autologous serum (autologous serum skin test, ASST) causes a wheal-and-flare reaction (19). This phenomenon mirrors the activation of mast cells and basophils as well as of the complement cascade by these autoantibodies. Experimental and clinical findings have supported an autoimmune origin in about 30–50% of patients affected with CSU (12, 13). Also other autoantibodies are involved in CSU pathogenesis such as autoantibodies to CD23, the low-affinity IgE receptor (FcεRII), which contribute to eosinophil activation and mast cell degranulation (14, 25). The possible autoimmune nature of CSU is further supported by the association with other autoimmune diseases, notably thyroiditis, and the increased frequency of HLA DRB1^*^04 (DR4) (26).

Inflammation is involved in the pathophysiology CSU, as demonstrated by the increase in different markers of inflammation, such as matrix metalloproteinase, serum C-reactive protein, IL-6, IL-6 soluble receptor, eosinophil cationic protein (ECP), TNF-α, complement, and others, which are related to CU activity (15–17, 27). On the contrary, adipokines that affect immune responses and exert an anti-inflammatory effect, such as lipocalin 2, have a negative association with CU activity (18).

Coagulation involvement in the pathogenesis of CSU is supported by many lines of evidence (28–33). The original observation that the autologous plasma skin test (APST) may score positive in some ASST-negative patients (29) has given rise to investigation of the coagulation system in CSU patients. In a study that dates back over 10 years, some of us found that CSU patients have elevated plasma levels of prothrombin fragment F1+2, suggesting thrombin generation (29). In subsequent studies, we found that CSU patients show an activation of the tissue factor pathway of coagulation cascade by activated eosinophils (30). Immunohistochemical experiments showed tissue factor expression by eosinophils present in the inflammatory infiltrate of CSU skin lesions (31). These data highlight the relevance of eosinophils in CSU as a source of tissue factor, in accordance with studies showing that eosinophils store tissue factor and transfer it to their cell membrane during activation (34). Eosinophils may be activated directly by autoantibodies directed against the FcεRII CD23 antigen (14) or secondarily to the activation of mast cells by anti-FcεRI and anti-IgE autoantibodies (24). The activation of the tissue factor pathway of coagulation results, in turn, in the generation of thrombin which, in experimental models, has been shown to induce edema through an increase in vascular permeability directly by acting on endothelial cells (35) and indirectly by generating C5a (36, 37), by triggering mast cell degranulation (38–41) and by releasing inflammatory mediators (42, 43). Pro-inflammatory cytokines, such as IL-6 and TNF-α, induce the expression of tissue factor (3, 44), sealing the mutual activation of the two systems, i.e., coagulation and inflammation. The complexes between tissue factor, activated factor VII (FVIIa) and FVa+FXa also activate mast cells via PAR-2, thus amplifying the activation of these cells in CSU (40). On the other hand, mast cell-derived tryptase can induce thrombin generation through a direct activation of prothrombin (45), thus constituting an amplification loop. It must be taken into account also an opposite effect of mast cells on inflammatory response. In fact, recent studies showed that mast cells can also limit thrombin-induced immediate skin inflammatory responses by releasing substances able to reduce the activity of thrombin, for example proteases, like mast cell protease 4 (MCPT4) (46). During severe exacerbations of the disease, CSU patients show marked increase of plasmatic markers of thrombin generation, like prothrombin fragment F1 + 2, FVIIa and thrombin-antithrombin complex (47–49), as well as of fibrinolysis (24, 31, 50). The increase of D-dimer, marker of fibrinolysis, is associated with disease severity (24) and its level resulted higher when compared with that of patients affected with psoriasis (51). Interestingly, a study detected the increase of D-dimer and fibrinogen/fibrin degradation products (FDP), produced by fibrinolysis of either stabilized or non-stabilized fibrin, in patients with CSU, but not in healthy individuals or in patients with inducible type of urticaria (52). Finding that the increase in plasma markers of thrombin generation and fibrinolysis parallels plasma C-reactive protein (CRP) levels further remarks the close link between coagulation activation and inflammation in CSU pathogenesis (53). The interplay between inflammatory and coagulation/fibrinolysis factors in CSU may lead to maintenance and amplification of urticarial inflammation. Both the activation of coagulation and fibrinolysis decrease till complete normalization during remission and biomarker levels return to normal values (31, 49, 54, 55). A limited number of studies investigated the role of platelets in CSU, with no univocal data (56). Platelet activation was found to occur in some skin inflammatory disorders, such as atopic dermatitis and psoriasis (57, 58). Based on these findings, platelets are regarded as a possible link between chronic inflammatory and pro-coagulant states (59). It may be hypothesized that platelet activation may be one of the triggering factors of the coagulation cascade in CSU too, leading to the activation of histamine-releasing effector cells. However, current evidence indicates that the simple determination of platelet indices is not reliable and lacks useful implications in the clinical practice (56).

The activation of the coagulation cascade in CSU pathogenesis may have clinical effects not only in eliciting skin lesions, namely wheal eruption, but also at systemic level. The most important potential clinical consequence of the hypercoagulable state in CSU patients is an increased thrombotic risk (25). In spite of this, patients with CSU are not reported to have an increased risk for thrombotic events. A possible explanation is that the activation of coagulation occurs mostly extravascularly and can be efficiently counteracted by coagulation inhibitors and fibrinolysis. Other potential clinical implications of the recognized involvement of coagulation in the pathogenesis of CSU are the possible therapeutic efficacy of pharmacological agents that interfere with the coagulation pathway. A few reports indicate that heparin, an anticoagulant which potentiates the effect of antithrombin, may be effective in treating CSU (60, 61). An anticoagulant/anti-fibrinolytic therapy, namely nadroparin and tranexamic acid, has been found effective in some patients with refractory CSU and elevated D-dimer levels (62). Moreover, warfarin, an oral anti-vitamin K drug, induced a good response in patients with CU unresponsive to antihistamines in a double-blind, placebo controlled study (63). With reference to further clinical implications of the activation of coagulation and fibrinolysis, elevated D-dimer plasma level has been found to be associated both with a poor response to antihistamines (64) and with a limited response to cyclosporine treatment (65). It is worthy of note that D-dimer plasma levels were observed to parallel the clinical response to omalizumab treatment, dropping in responders and remaining unchanged in non-responders (20, 66). Furthermore, elevated D-dimer plasma levels seem to be a predictive marker of prompt and complete response to the anti-IgE monoclonal antibody (67). It may be assumed that in a subset of CSU patients, the activation of the coagulation cascade has a key role in the pathogenesis of this disorder. In a recent study, the treatment with omalizumab induced a significant decrease in WBC count, platelet count, neutrophil count, ratio of platelet to lymphocyte (PLR), ratio of neutrophil to lymphocyte (NLR) and CRP level and a significant increase in mean platelet volume (MPV) and eosinophil count (68). Taken together, these data suggest that in CSU patients omalizumab may be effective not only antagonizing IgE but also affecting a number of other pathways (69). In particular, it can exert inhibitory effects on inflammation and coagulation cascade, further confirming the strict interaction between immunity, inflammation, and coagulation.

The “autoallergic” mechanism, which has been recently identified, is mediated by specific IgE to different allergens, including double-strained DNA (21), thyroperoxidase (22), IL-24 (23), tissue factor, and thyroglobulin (20). IgE anti-autoallergens seem to cause “autoallergic” mast cell degranulation. In particular, the elevated specific IgE antibodies to tissue factor have been demonstrated to be functionally able to mediate the release of leukotriene C4 by tissue factor-stimulated peripheral basophils (20). Tissue factor, the main initiator of coagulation, is overexpressed in CSU lesional skin (32) where it can activate coagulation and is also accessible to mast cell-bound IgE. Thus, IgE anti-tissue factor could cause “autoallergic” mast cell degranulation, linking the autoallergic and coagulation activation pathways. The presence of autoreactive IgE against several targets in patients with CSU might explain, at least in part, the clinical success of the humanized monoclonal anti-IgE antibody omalizumab.

## Angioedema

Angioedema refers to a circumscribed, non-itchy edema of the subcutaneous tissues mostly involving lips, face, neck and extremities and/or submucosal tissues affecting oral cavity, larynx and gut (70). It typically lasts from many hours to 3 days before the tissue returns to normal. Angioedema is life-threatening if it occurs in the upper airways and can be very painful if it occurs in the gastrointestinal tract (70). Wheals and angioedema often coexist but may also present alone (71). Most of these conditions are mainly mediated by histamine and other pro-inflammatory mediators released by mast cells and basophils (72). Overall, angioedema can be caused by allergies, inherited or acquired deficiencies of C1-inhibitor protein, or drug reactions. In many cases its pathophysiology remains undetermined (54). Among the vasoactive agents involved in mediating swelling attacks in angioedema, histamine, released by mast cells or basophils, has a prominent role, especially in allergic reactions and in cases of associated urticaria (73). Anti-histamines are effective agents in these forms. However, there are some subtypes of angioedema that do not respond to anti-histamine, such as angioedema due to C1-inhibitor deficiency, angioedema due to ACE inhibitors and a rate of idiopathic angioedema (54). Bradykinin is a recognized mediator of the increased vascular permeability in angioedema. Bradykinin is a vasoactive peptide acting on specific receptors such as B2 receptors that are constitutively present on the endothelium and B1 receptors that are expressed during inflammatory reactions. Bradykinin receptor signaling induces peripheral vasodilatation, enhancement of vascular permeability and subsequent vascular leakage. Bradykinin is the end-product of the contact activation system. This enzymatic cascade circulates in the plasma and consists of factor XII (FXII), plasma prekallikrein (PPK), and high molecular weight kininogen (HK). This system is linked to the intrinsic coagulation system via factor XI (FXI). Animal models showed that production of bradykinin in plasma is ~50% dependent on FXII (74). This suggests the presence of alternative pathways generating bradykinin *in vivo*. The endothelial cell-derived factors *heat shock protein 90* (HSP90) and prolylcarboxypeptidase may be involved in FXII-independent release of bradykinin (75).

Bradykinin was first identified as a mediator for hereditary angioedema (HAE) (76, 77). The genetic form of C1-inhibitor deficiency is due to mutations in one of the two alleles of the C1-inhibitor gene, which leads to either reduced plasma protein levels [hereditary angioedema [HAE] type I] or normal levels but reduced protein function (HAE type II) (78). HAE type III is a rare subtype of HAE that is not connected with C1-inhibitor deficiency but with a dysregulation of the contact (plasma kallikrein-bradykinin) system (79). In a subset of patients with HAE with normal C1-inhibitor, a gain-of-function mutation in FXII has been identified (80). The acquired form of C1-inhibitor deficiency is known as acquired angioedema and is due to C1-inhibitor consumption associated with the presence of anti-C1-inhibitor autoantibodies and/or lymphoproliferative disorders (81). Bradykinin involvement in angioedema pathogenesis is not limited to hereditary forms. Bradykinin is a key mediator in patients with acquired C1-inhibitor deficiency due to underlying auto-immune or lymphoproliferative diseases (82) as well as in those treated with anti-hypertensive drugs that inhibit bradykinin breakdown, such as angiotensin-converting enzyme inhibitors (83). It is also possible that bradykinin may play a supportive role in forms of angioedema that are currently classified as “histaminergic” (84, 85). C1-inhibitor deficiency involves different biological systems that interplay during angioedema attacks. In fact, C1 inhibitor is a serine protease inhibitor (serpin) that blocks the activity of (i) C1r and C1s in the complement system, (ii) factor XII and kallikrein in the contact system, (iii) factor XI and thrombin in the coagulation system, and (iv) tissue plasminogen activator and plasmin in the fibrinolytic system (86). A deficiency in C1 inhibitor results in the hyperactivation of the contact system (87, 88), which leads to the generation of bradykinin. Furthermore, C1-inhibitor deficiencies activate the complement (89) as well as coagulation (90, 91) and fibrinolysis systems (92, 93). Thus, the unregulated activation of coagulation leads to the generation of thrombin, which can potentiate the vasoactive effect of bradykinin both directly (35, 94) and by releasing fibrinopeptides, which enhance the effects of kinins (95). It may be argued that in angioedema due to C1-inhibitor deficiency, thrombin acts synergistically with other vasoactive substances released by the concomitant activation of contact system, complement, or mast cells leading in turn to increased vasopermeability. Plasmin has been assumed to act as a main trigger for contact system activation and bradykinin production in the pathogenesis of most forms of HAE and specific forms of non-hereditary angioedema (96, 97). Indeed, several clinical observations supported the relevance of plasmin as a natural FXII activator and evidence for plasmin-dependent bradykinin generation as a cause of angioedema during treatment with fibrinolytic agents is accumulating (73). Consistent with this hypothesis, complexes of plasmin with its inhibitor α2-antiplasmin are elevated during attacks of HAE due to C1-inhibitor deficiency, as are the levels of markers of ongoing fibrinolysis, like D-dimer (98). Similarly to CSU, in patients with angioedema there is lack of prothrombotic features (55, 99). Therefore, it may be assumed that in angioedema, factor XII–driven contact system starts inflammatory mechanisms via the bradykinin-producing kallikrein-kinin system, without procoagulant effects (35). Alternatively, it has been proposed that the extreme vascular leakage may move the plasma coagulation factors into the extravascular space, triggering coagulation in the absence of vascular injury or intravascular thrombi (73). Therapy targeting the contact system has been successful in HAE, strongly supporting that angioedema is mediated via bradykinin production (73). Anti-fibrinolytic therapy, mainly tranexamic acid, has been used as prophylactic therapy for HAE attacks for some decades (100).

## Bullous Pemphigoid

**Figure 2:**
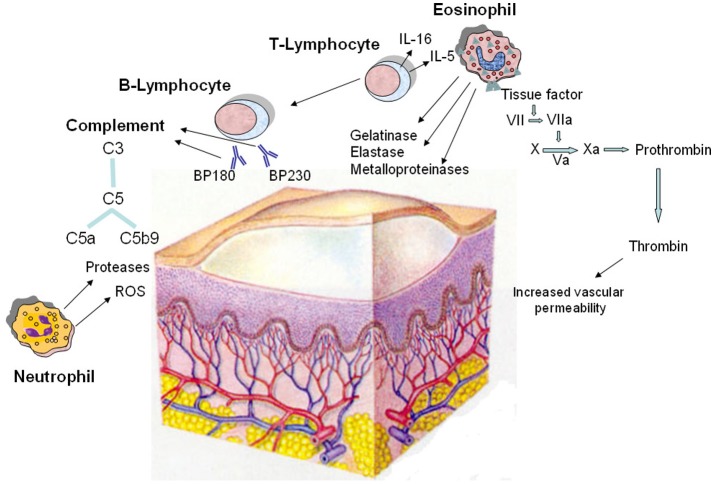
Pathomechanisms of the blister formation in bullous pemphigoid including the involvement of coagulation activation. The dermo-epidermal detachment is due to the interaction of autoantibodies with two hemidesmosomal antigens (BP180 and BP230), followed by complement activation and leukocyte infiltration. Autoreactive T lymphocytes cooperate with B lymphocytes in the autoantibody production; in addition, they release cytokines, most notably IL-5 and IL-16, and other soluble factors responsible for the recruitment and activation of eosinophils. In lesional skin, eosinophils produce and release metalloproteinases, elastase, and gelatinase which contribute to tissue damage. Moreover, eosinophils strongly express tissue factor, which is the main initiator of the coagulation cascade (factors VII, X, VIII, V, and prothrombin) leading to generation of thrombin. This last increases the permeability of blood vessels, amplifying the inflammatory network. Neutrophils contribute in the pathogenesis of bullous pemphigoid by releasing reactive oxygen species (ROS) and proteases. Finally, complement is activated upon binding of the pathogenic autoantibodies to their autoantigens.

Bullous pemphigoid (BP) is an autoimmune blistering disease that mainly affects the elderly and carries a high risk of death (101, 102), mostly due to sepsis and cardiovascular events (103). Either comorbidities or immunosuppressive treatments seem to concur on BP mortality (104). Albeit rare, it is the most common autoimmune blistering disease in Western countries (105). It involves the skin and rarely the mucous membranes and is characterized by the presence of tense blisters usually surrounded by erythematous-edematous, urticaria-like plaques (106, 107). BP has a chronic relapsing evolution, with spontaneous exacerbations and remissions (108). The pathophysiology of BP is linked to the presence of circulating IgG autoantibodies directed against two antigens, BP180 and BP230, which are components of junctional adhesion complexes called hemidesmosomes that promote dermoepidermal cohesion (109, 110) with the contributory role of other pathomechanisms (Figure 2). BP180 (type XVII collagen) autoantibodies have been demonstrated to be directly pathogenic by triggering an inflammatory cascade. Inflammatory cells, particularly autoreactive T cells and eosinophils, produce and release a number of cytokines and soluble factors that amplify and maintain tissue damage, which ultimately leads to subepidermal blister formation (111). During acute phase of BP, autoreactive T helper (Th) 1, Th2, and Th17 lymphocytes cooperatively play a role in the development of the disease process (112–114). Although the role of Th17 cells in human BP is not completely defined, recent experimental data showed that IL17A-deficient mice are protected against autoantibody-induced BP, and pharmacological inhibition of lL-17A reduces the induction of BP (115). Moreover, a dysfunction of both T and B regulatory cells, whose immunosurveillance action is critical in preventing autoimmunity, was observed in lesional skin of BP patients (116, 117). The involvement of many cellular players in the BP inflammatory process is supported by elevated serum levels of activation markers, including molecules released by T cells upon their stimulation, molecules involved in B-cell maturation, granule proteins liberated from activated mast cells, neutrophils and eosinophils and adhesion molecules indicative of neutrophil and platelet activation. Complement activation is considered to be critical for blister formation too. Indeed, complement activation by autoantibodies *ex vivo* as measured by the complement-fixation assay in serum was correlated with the clinical disease activity in patients with BP (118). Experimental data in murine models demonstrate that complement-dependent and -independent mechanisms coexist in blister formation. On the basis of knock out mice and pharmacological inhibition studies, the activated 5th component of complement (C5a) appears to be involved, and its receptor 1 (C5aR1) seems to be important during the early phase of the disease while its receptor 2 (C5aR2) seems to be protective. Once the skin inflammation has fully developed, release of reactive oxygen species and proteases from neutrophils and macrophages may become independent of complement (119). Several studies showed that the coagulation cascade is activated in BP and correlates with the severity of the disease (25, 120–123). In particular, coagulation activation markers have been found to be increased at both local level, i.e., in blister fluid, and systemically, i.e., in plasma (123). It has been hypothesized that the activation of blood coagulation is induced by the inflammatory response underlying BP pathogenesis. In this regard, eosinophils are highly represented in the inflammatory infiltrate of the lesional skin and their levels are often increased in peripheral blood (123–125). Elevated concentrations of secretory granules, such as eosinophil cationic protein (ECP) (126, 127) as well as increased levels of IL5, the main cytokine involved in eosinophil biology (128, 129), and IL16, a chemotactic factor for eosinophils (130), in blister fluid and sera of patients with BP confirm the involvement of eosinophils in the pathogenesis of this disease. Eosinophils are recognized to concur in BP elicitation by producing and releasing matrix metalloproteinase (MMP)-9, which plays a key role in degrading BP180 and cleaving the dermal-epidermal junction (131). Release of elastase and gelatinase, namely 92 kDa gelatinase, may further contribute to tissue damage and blister formation (132, 133). Eosinophil degranulation was demonstrated not only in fully developed blisters, but also at the earliest stages of blister development and urticarial lesions of BP (134, 135). Moreover, eosinophils have been postulated as potential intermediates between anti-BP180 IgE autoantibodies, whose pathogenic role in BP has increasingly recognized in recent years, and dermo-epidermal junction separation (136, 137). High-affinity IgE receptors (FcεRI) that are highly expressed on eosinophils in BP patients could enhance the capability of eosinophils to bind IgE and thus influence their subsequent degranulation (138). In recent reports, the monoclonal anti-IgE antibody omalizumab resulted effective in treating patients affected with BP, despite ongoing high levels of anti-skin IgG antibodies (139, 140). This provides further evidence of an independent role for autoreactive IgE-mediated inflammation in BP skin lesion development. Eosinophils are also involved in itch induction, mainly due to the release of IL-31 (141, 142). IL31 exerts a chief role in itch, by activating endothelin-1 responsive neurons and by increasing the release of brain natriuretic peptide (BNP), a central mediator of itch (143, 144). With specific reference to the activation of the coagulation cascade in BP, it correlates with eosinophilia other than severity of the disease, thus indicating that eosinophils play a pivotal role in this process (123). Immunohistochemistry showed that eosinophils strongly express TF in lesional skin, as confirmed also by co-localization studies (120, 123). Eosinophils are a major intravascular location for TF storage and exposure. On the other hand, TF facilitates the early transendothelial migration of eosinophils (34). Consistent with these findings, it may be supposed that extrinsic blood coagulation initiated via TF expressed by eosinophils contributes to local inflammation and blister formation (145). In fact, TF plays an important role in the inflammatory process, in addition to its well-documented prothrombotic properties (34). In BP patients, increased skin expression of adhesins and MMPs has been considered an effect of TF action (146). TF is a recognized factor connecting the immune system with coagulation system. Pro-inflammatory cytokines, such as TNF and IL-6, which are increased in BP, induce TF expression (25), as found also in other, very heterogeneous inflammatory conditions (147–149). Activation of the extrinsic coagulation pathway generates thrombin that increases the permeability of blood vessels (150, 151). The presence of thrombin may play a direct role in the pathogenesis of BP by increasing vascular permeability, thus favoring the transendothelial migration of inflammatory cells and their accumulation in the skin. Activated proteases, in turn, act on PARs and induce the expression of various pro-inflammatory cytokines, and this cross-talk between inflammation and coagulation amplifies and maintains the activation of both systems (123). It is noteworthy that BP patients have high levels of coagulation activation markers, such as prothrombin fragment F1+2 (indicating thrombin generation) and D-dimer (indicating fibrin degradation), in plasma samples other than in blister fluid (120, 121, 123). During the disease remission, blood concentration of coagulation activation markers returns to normal (49, 145). Moreover, the concentration of the prothrombin fragment F1+2 correlates with the concentration of immunoglobulins directed against the BP180 antigen (120). All together, these findings clearly indicate that in BP patients there may be an activation of coagulation also at systemic level. A recent study has shown that plasma levels of prothrombotic markers are higher in BP patients when compared with patients affected with CSU (49). In pathological states, especially in inflammatory disorders, the balance between the coagulation and fibrinolysis might be deteriorated (152, 153). It is well known that the inflammatory response inhibits fibrinolysis. The results of a previous study on a group of patients with active BP showed that fibrinolysis is inhibited, due mainly to an increase in the plasma levels of plasminogen activator inhibitor type 1 (PAI-1) activity and antigen (154). The most important clinical consequence of the hypercoagulable state related to both coagulation activation and fibrinolysis inhibition in BP patients is an increased thrombotic risk. It has been consistently reported that the risk of thrombosis is increased in patients with BP (155, 156) and we have found an annual incidence of venous thrombosis of 8% (120), undoubtedly higher than that observed in the general elderly population (157). Langan et al. investigated the main acute medical conditions associated with BP, finding an increased risk for pulmonary embolism (158). A recent multicenter cohort study has shown that the risk of developing venous thromboembolism (VTE) is increased 4-fold in patients with BP as compared with the general population of same age and sex (159). More specifically, the VTE risk increased up to 15 times during the acute phase of the disease, thus indicating a close link between the inflammatory state occurring in active BP and thrombosis. Interestingly, the VTE risk dropped to 1.5 times during clinical remission. This latter finding indicates that a good control of the disease also impacts the occurrence of thrombotic events. This study highlighted in a large cohort of BP patients the close connection between the inflammatory state and risk of VTE. This further emphasizes the tight interplay between inflammation and coagulation cascade. In general, the cardiovascular risk in BP patients may be influenced not only by the inflammatory state but also by the treatment with systemic corticosteroids and immunosuppressive agents (160).

The high thrombotic tendency found in patients with BP, especially during the acute phase of the disease, implies some practical considerations. Firstly, one may wonder whether adding VTE prophylaxis to the immunosuppressive treatments could affect the thrombotic risk of BP patients. Clinical trials on efficacy and safety of antithrombotic drugs administered in the acute phase of the disease could provide some insight into their clinical relevance.

## Conclusions

Coagulation and fibrinolysis activation markers are elevated in many inflammatory conditions, including not only a wide spectrum of systemic diseases of different pathogenesis such as rheumatoid arthritis, inflammatory bowel diseases, and sepsis but also autoimmune or immune-mediated cutaneous disorders. The activation of coagulation in autoimmune skin disorders may have two main consequences: on one hand, a local pathogenic role in inducing the skin lesions and on the other hand a systemic role in increasing the thrombotic risk. Concerning the pathophysiology of the skin lesions, thrombin contributes to increase endothelial vascular permeability, thus amplifying the inflammatory network in CU, angioedema and BP. In the skin microenvironment of CU and BP, eosinophils express tissue factor, which can activate coagulation right there, with the generation of vascular permeability mediators. Concerning the systemic implications of the coagulation involvement, several data indicate an increased thrombotic risk in BP. In contrast, no data are available concerning the incidence of thrombotic complications in patients with CU and angioedema. The retrospective evaluation of a large cohort of patients with BP has shown an increased incidence of venous thrombosis. Although most BP patients are elderly, the incidence of venous thrombosis in this kind of patients appears to be significantly increased as compared to the age-matched population of otherwise healthy subjects. BP is the prototype of autoimmune blistering disorder mediated by autoantibodies but in which an important physiopathological role is played by eosinophils. The intense skin inflammatory infiltrate is in fact characterized by the presence of eosinophils together with autoreactive CD4+ T lymphocytes and a few other inflammatory cells. The increased incidence of thrombosis in autoimmune skin diseases raises the question of the indication of anticoagulant treatment particularly in the presence of other cardiovascular risk factors.

## Author Contributions

MC, AM, and AB designed and drafted the manuscript. MC, AM, and AB made the literature search. MC, AM, AB, and SG discussed the topics of the manuscript and approved the final version.

### Conflict of Interest Statement

The authors declare that the research was conducted in the absence of any commercial or financial relationships that could be construed as a potential conflict of interest.

## References

[1] Opal SM. Phylogenetic and functional relationships between coagulation and the innate immune response. Crit Care Med. (2000) 28:S77–80. 10.1097/00003246-200009001-00017 11007204

[2] Esmon CT. Crosstalk between inflammation and thrombosis. Maturitas. (2008) 61:122–31. 10.1016/j.maturitas.2008.11.008 19437587

[3] Levi M van der Poll T. Two-way interactions between inflammation and coagulation. Trends Cardiovasc Med. (2005) 15:254–9. 10.1016/j.tcm.2005.07.004 16226680

[4] Chu AJ. Tissue factor mediates inflammation. Arch Biochem Biophys. (2005) 440:123–32. 10.1016/j.abb.2005.06.005 16036212

[5] Dhar J Andersen J Essenmacher L Ager J Bentley G Sokol R. Thrombophilic patterns of coagulation factors in lupus. Lupus. (2009) 18:400–6. 10.1177/0961203308097566 19318391

[6] Kern A Barabás E Balog A Burcsár S Kiszelák M Vásárhelyi B. Characterization of the thrombin generation profile in systemic lupus erythematosus. Physiol Int. (2017) 104:35–41. 10.1556/2060.104.2017.1.5 28361572

[7] Ingegnoli F Fantini F Favalli EG Soldi A Griffini S Galbiati V. Inflammatory and prothrombotic biomarkers in patients with rheumatoid arthritis: effects of tumor necrosis factor-alpha blockade. J Autoimmun. (2008) 31:175–9. 10.1016/j.jaut.2008.07.002 18707846

[8] Łukasik ZM Makowski M Makowska JS. From blood coagulation to innate and adaptive immunity: the role of platelets in the physiology and pathology of autoimmune disorders. Rheumatol Int. (2018) 38:959–74. 10.1007/s00296-018-4001-9 29492586 PMC5954012

[9] Borensztajn K Peppelenbosch MP Spek CA. Coagulation factor Xa signaling: the link between coagulation and inflammatory bowel disease? Trends Pharmacol Sci. (2009) 30:8–16. 10.1016/j.tips.2008.10.007 19058861

[10] Magro F Soares JB Fernandes D. Venous thrombosis and prothrombotic factors in inflammatory bowel disease. World J Gastroenterol. (2014) 20:4857–72. 10.3748/wjg.v20.i17.4857 24803797 PMC4009517

[11] Zuberbier T Aberer W Asero R Bindslev-Jensen C Brzoza Z Canonica GW The EAACI/GA^2^LEN/EDF/WAO Guideline for the definition, classification, diagnosis, and management of urticaria: the 2013 revision and update. Allergy. (2014) 69:868–87. 10.1111/all.12313 24785199

[12] Greaves MW. Pathology and classification of urticaria. Immunol Allergy Clin North Am. (2014) 34:1–9. 10.1016/j.iac.2013.07.009 24262685

[13] Saini SS. Chronic spontaneous urticaria: etiology and pathogenesis. Immunol Allergy Clin North Am. (2014) 34:33–52. 10.1016/j.iac.2013.09.012 24262688 PMC11218737

[14] Puccetti A Bason C Simeoni S Millo E Tinazzi E Beri R. In chronic idiopathic urticaria autoantibodies against Fc epsilonRII/CD23 induce histamine release via eosinophil activation. Clin Exp Allergy. (2005) 35:1599–607. 10.1111/j.1365-2222.2005.02380.x 16393326

[15] Kasperska-Zajac A Grzanka A Damasiewicz-Bodzek A. IL-6 transsignaling in patients with chronic spontaneous urticaria. PLoS ONE. (2015) 10:e0145751. 10.1371/journal.pone.0145751 26699882 PMC4689405

[16] Ye YM Jin HJ Hwang EK Nam YH Kim JH Shin YS. Co-existence of chronic urticaria and metabolic syndrome: clinical implications. Acta Derm Venereol. (2013) 93:156–60. 10.2340/00015555-1443 22948845

[17] Kolkhir P Altrichter S Hawro T Maurer M. C-reactive protein is linked to disease activity, impact, and response to treatment in patients with chronic spontaneous urticaria. Allergy. (2018) 73:940–8. 10.1111/all.13352 29130488

[18] Trinh HK Pham DL Ban GY Lee HY Park HS Ye YM. Altered systemic adipokines in patients with chronic urticaria. Int Arch Allergy Immunol. (2016) 171:102–10. 10.1159/000452626 27902979

[19] Cugno M Marzano AV Asero R Tedeschi A. Activation of blood coagulation in chronic urticaria: pathophysiological and clinical implications. Intern Emerg Med. (2010) 5:97–101. 10.1007/s11739-009-0333-5 19949893

[20] Cugno M Asero R Ferrucci S Lorini M Carbonelli V Tedeschi A. Elevated IgE to tissue factor and thyroglobulin are abated by omalizumab in chronic spontaneous urticaria. Allergy. (2018) 73:2408–11. 10.1111/all.13587 30076634

[21] Hatada Y Kashiwakura J Hayama K Fujisawa D Sasaki-Sakamoto T Terui T. Significantly high levels of anti-dsDNA immunoglobulin E in sera and the ability of dsDNA to induce the degranulation of basophils from chronic urticaria patients. Int Arch Allergy Immunol. (2013) 161:154–8. 10.1159/000350388 23711867

[22] Altrichter S Peter HJ Pisarevskaja D Metz M Martus P Maurer M. IgE mediated autoallergy against thyroid peroxidase–a novel pathomechanism of chronic spontaneous urticaria? PLoS ONE. (2011) 6:e14794. 10.1371/journal.pone.0014794 21532759 PMC3075251

[23] Schmetzer O Lakin E Topal FA Preusse P Freier D Church MK. IL-24 is a common and specific autoantigen of IgE in patients with chronic spontaneous urticaria. J Allergy Clin Immunol. (2018) 142:876–82. 10.1016/j.jaci.2017.10.035 29208545

[24] Tedeschi A Kolkhir P Asero R Pogorelov D Olisova O Kochergin N. Chronic urticaria and coagulation: pathophysiological and clinical aspects. Allergy. (2014) 69:683–91. 10.1111/all.12389 24673528

[25] Cugno M Tedeschi A Asero R Meroni PL Marzano AV. Skin autoimmunity and blood coagulation. Autoimmunity. (2010) 43:189–94. 10.3109/08916930903293086 19883336

[26] O'Donnell BF O'Neill CM Francis DM Niimi N Barr RM Barlow RJ. Human leucocyte antigen class II associations in chronic idiopathic urticaria. Br J Dermatol. (1999) 140:853–8. 10.1046/j.1365-2133.1999.02815.x 10354022

[27] Kessel A Bishara R Amital A Bamberger E Sabo E Grushko G. Increased plasma levels of matrix metalloproteinase-9 are associated with the severity of chronic urticaria. Clin Exp Allergy. (2005) 35:221–5. 10.1111/j.1365-2222.2005.02168.x 15725195

[28] Asero R Tedeschi A Marzano AV Cugno M. Chronic urticaria: a focus on pathogenesis. F1000Res. (2017) 6:1095. 10.12688/f1000research.11546.1 28751972 PMC5506533

[29] Asero R Tedeschi A Riboldi P Cugno M. Plasma of chronic urticaria patients shows signs of thrombin generation and its intradermal injection causes wheal-and-flare reactions much more frequently than autologous serum. J Allergy Clin Immunol. (2006) 117:1113–7. 10.1016/j.jaci.2005.12.1343 16675340

[30] Asero R Tedeschi A Coppola R Griffini S Paparella P Riboldi P. Activation of the tissue pathway of blood coagulation in patients with chronic urticaria. J Allergy Clin Immunol. (2007) 119:705–10. 10.1016/j.jaci.2006.08.043 17204316

[31] Asero R Tedeschi A Riboldi P Griffini S Bonanni E Cugno M. Severe chronic urticaria is associated with elevated plasma levels of D-dimer. Allergy. (2008) 63:176–80. 10.1111/j.1398-9995.2007.01514.x 17961199

[32] Cugno M Marzano AV Tedeschi A Fanoni D Venegoni L Asero R. Expression of tissue factor by eosinophils in patients with chronic urticaria. Int Arch Allergy Immunol. (2009) 148:170–4. 10.1159/000155748 18802362

[33] Takeda T Sakurai Y Takahagi S Kato J Yoshida K Yoshioka A. Increase of coagulation potential in chronic spontaneous urticaria. Allergy. (2011) 66:428–33. 10.1111/j.1398-9995.2010.02506.x 21083568

[34] Moosbauer C Morgenstern E Cuvelier SL Manukyan D Bidzhekov K Albrecht S. Eosinophils are a major intravascular location for tissue factor storage and exposure. Blood. (2007) 109:995–1002. 10.1182/blood-2006-02-004945 17003379

[35] Schaeffer RC Gong F Bitrick MS Smith TL. Thrombin and bradykinin initiate discrete endothelial solute permeability mechanisms. Am J Physiol. (1993) 264:1798–809. 10.1152/ajpheart.1993.264.6.H1798 8322908

[36] Asero R Riboldi P Tedeschi A Cugno M Meroni P. Chronic urticaria: a disease at a crossroad between autoimmunity and coagulation. Autoimmun Rev. (2007) 7:71–6. 10.1016/j.autrev.2007.08.002 17967729

[37] Huber-Lang M Sarma JV Zetoune FS Rittirsch D Neff TA McGuire SR. Generation of C5a in the absence of C3: a new complement activation pathway. Nat Med. (2006) 12:682–7. 10.1038/nm1419 16715088

[38] Vliagoftis H. Thrombin induces mast cell adhesion to fibronectin: evidence for the involvement of protease activated receptor-1. J Immunol. (2002) 169:4551–8. 10.4049/jimmunol.169.8.4551 12370392

[39] Razin E Marx G. Thrombin-induced degranulation of cultured bone marrow derived mast cells. J Immunol. (1984) 133:3282–5. 6208276

[40] Dugina TN Kiseleva EV Glusa E Strukova SM. Activation of mast cells induced by agonists of proteinase-activated receptors under normal conditions and during acute inflammation in rats. Eur J Pharmacol. (2003) 471:141–7. 10.1016/S0014-2999(03)01752-7 12818702

[41] Yanase Y Takahagi S Hide M. Chronic spontaneous urticaria and the extrinsic coagulation system. Allergol Int. (2018) 67:191–4. 10.1016/j.alit.2017.09.003 28993062

[42] Cirino G Cicala C Bucci MR Sorrentino L Maranganore JM Stone SR Thrombin functions as an inflammatory mediator through activation of its receptors. J Exp Med. (1996) 183:821–7. 10.1084/jem.183.3.821 8642286 PMC2192352

[43] Tedeschi A Asero R Lorini M Marzano AV Cugno M Plasma levels of matrix metalloproteinase-9 in chronic urticaria patients correlate with disease severity and C-reactive protein but not with circulating histamine-releasing factors. Clin Exp Allergy. (2010) 40:875–81. 10.1111/j.1365-2222.2010.03473.x 20214668

[44] van der Poll T de Boer JD Levi M. The effect of inflammation on coagulation and vice versa. Curr Opin Infect Dis. (2011) 24:273–8. 10.1097/QCO.0b013e328344c078 21330919

[45] Kido H Fukusen N Katunuma M Morita T Iwanaga S. Tryptase from rat mast cells converts bovine prothrombin to thrombin. Biochem Biophys Res Commun. (1985) 132:613–9. 10.1016/0006-291X(85)91177-5 3904753

[46] Suender CA Leist M Åbrink M Valentin P Geldmacher A Steinhoff M. Mast cells are critical for the limitation of thrombin-induced skin inflammation. Exp Dermatol. (2018) 27:50–7. 10.1111/exd.13407 28787094

[47] Wang F Tang H Xu JH Kang KF. Activation of the blood coagulation cascade is involved in patients with chronic urticaria. J Allergy Clin Immunol. (2009) 123:972–3. 10.1016/j.jaci.2009.01.039 19251312

[48] Fujii K Usuki A Kan-No Y Ohgou N. Elevation of circulating thrombin-antithrombin III complex and fibrin degradation products in urticaria. A laboratory finding unrelated to intravascular coagulopathy. J Dermatol. (2008) 35:308–10. 10.1111/j.1346-8138.2008.00474.x 18477235

[49] Cugno M Tedeschi A Borghi A Bucciarelli P Asero R Venegoni L. Activation of blood coagulation in two prototypic autoimmune skin diseases: a possible link with thrombotic risk. PLoS ONE. (2015) 10:e0129456. 10.1371/journal.pone.0129456 26057532 PMC4461280

[50] Triwongwaranat D Kulthanan K Chularojanamontri L Pinkaew S. Correlation between plasma D-dimer levels and the severity of patients with chronic urticaria. Asia Pac Allergy. (2013) 3:100–5. 10.5415/apallergy.2013.3.2.100 23667833 PMC3643060

[51] Criado PR Antinori LC Maruta CW Reis VM. Evaluation of D-dimer serum levels among patients with chronic urticaria, psoriasis and urticarial vasculitis. An Bras Dermatol. (2013) 88:355–60. 10.1590/abd1806-4841.20131532 23793207 PMC3754365

[52] Takahagi S Mihara S Iwamoto K Morioke S Okabe T Kameyoshi Y. Coagulation/fibrinolysis and inflammation markers are associated with disease activity in patients with chronic urticaria. Allergy. (2010) 65:649e56. 10.1111/j.1398-9995.2009.02222.x 19845571

[53] Grzanka R Damasiewicz-Bodzek A Kasperska-Zajac A Interplay between acute phase response and coagulation/fibrinolysis in chronic spontaneous urticaria. Allergy Asthma Clin Immunol. (2018) 18:14 10.1186/s13223-018-0255-8 PMC6050720 30026764

[54] Khalaf AT Liu XM Sheng WX Tan JQ Abdalla AN. Efficacy and safety of desloratadine combined with dipyridamole in the treatment of chronic urticaria. J Eur Acad Dermatol Venereol. (2008) 22:487–92. 10.1111/j.1468-3083.2007.02511.x 18081747

[55] Cugno M Asero R Tedeschi A Lazzari R Marzano AV. Inflammation and coagulation in urticaria and angioedema. Curr Vasc Pharmacol. (2012) 10:653–8. 10.2174/157016112801784558 22272913

[56] Vena GA Cassano N Marzano AV Asero R. The role of platelets in chronic urticaria. Int Arch Allergy Immunol. (2016) 169:71–9. 10.1159/000444085 27035367

[57] Kasperska-Zajac A Brzoza Z Rogala B. Platelet function in cutaneous diseases. Platelets. (2008) 19:317–21. 10.1080/09537100802082249 18791936

[58] Tamagawa-Mineoka R Katoh N Kishimoto S. Platelet activation in patients with psoriasis: increased plasma levels of platelet-derived microparticles and soluble P-selectin. J Am Acad Dermatol. (2010) 62:621–6. 10.1016/j.jaad.2009.06.053 19962788

[59] Ghasemzadeh M Hosseini E. Platelet-leukocyte crosstalk: linking proinflammatory responses to procoagulant state. Thromb Res. (2013) 131:191–7. 10.1016/j.thromres.2012.11.028 23260445

[60] Meyer-De Schmid JJ Neuman A. Treatment of chronic urticaria with heparin. Bull Soc Fr Dermatol Syphiligr. (1952) 59:286–7. 12987986

[61] Chua SL Gibbs S. Chronic urticaria responding to subcutaneous heparin sodium. Br J Dermatol. (2005) 153:216–7. 10.1111/j.1365-2133.2005.06694.x 16029359

[62] Asero R Tedeschi A Cugno M. Heparin and tranexamic Acid therapy may be effective in treatment-resistant chronic urticaria with elevated d-dimer: a pilot study. Int Arch Allergy Immunol. (2010) 152:384–9. 10.1159/000292947 20203527

[63] Parslew R Pryce D Ashworth J Friedmann PS. Warfarin treatment of chronic idiopathic urticaria and angio-oedema. Clin Exp Allergy. (2000) 30:1161–5. 10.1046/j.1365-2222.2000.00857.x 10931124

[64] Asero R. D-dimer: a biomarker for antihistamine-resistant chronic urticaria. J Allergy Clin Immunol. (2013) 132:983–6. 10.1016/j.jaci.2013.04.037 23763979

[65] Asero R. Plasma D-dimer levels and clinical response to ciclosporin in severe chronic spontaneous urticaria. J Allergy Clin Immunol. (2015) 135:1401–3. 10.1016/j.jaci.2014.11.016 25542882

[66] Cugno M Genovese G Ferrucci S Casazza G Asero R Marzano AV. IgE and D-dimer baseline levels are higher in responders than nonresponders to omalizumab in chronic spontaneous urticaria. Br J Dermatol. (2018) 179:776–7. 10.1111/bjd.16593 29582427

[67] Asero R Marzano AV Ferrucci S Cugno M. D-dimer plasma levels parallel the clinical response to omalizumab in patients with severe chronic spontaneous urticaria. Int Arch Allergy Immunol. (2017) 172:40–4. 10.1159/000453453 28219067

[68] Acer E Kaya Erdogan H Yüksel Çanak çi N Saracoglu ZN The effect of omalizumab on hematological and inflammatory parameters in patients with chronic spontaneous urticaria. Cutan Ocul Toxicol. (2018) 10:1–4. 10.1080/15569527.2018.1495227 29969297

[69] Kaplan AP Giménez-Arnau AM Saini SS. Mechanisms of action that contribute to efficacy of omalizumab in chronic spontaneous urticaria. Allergy. (2017) 72:519–33. 10.1111/all.13083 27861988 PMC5915348

[70] Depetri F Tedeschi A Cugno M. Angioedema and emergency medicine: from pathophysiology to diagnosis and treatment. Eur J Intern Med. (2019) 59:8–13. 10.1016/j.ejim.2018.09.004 30220453

[71] Antia C Baquerizo K Korman A Bernstein JA Alikhan A. Urticaria: a comprehensive review: epidemiology, diagnosis, and work-up. J Am Acad Dermatol. (2018) 79:599–614. 10.1016/j.jaad.2018.01.020 30241623

[72] Borriello F Granata F Marone G. Basophils and skin disorders. J Invest Dermatol. (2014) 134:1202–10. 10.1038/jid.2014.16 24499736

[73] Hofman Z de Maat S Hack CE Maas C. Bradykinin: inflammatory product of the coagulation system. Clin Rev Allergy Immunol. (2016) 51:152–61. 10.1007/s12016-016-8540-0 27122021 PMC5025506

[74] Iwaki T Castellino FJ. Plasma levels of bradykinin are suppressed in factor XII-deficient mice. Thromb Haemost. (2006) 95:1003–10. 10.1160/TH06-03-0128 16732380

[75] Joseph K Tholanikunnel BG Bygum A Ghebrehiwet B Kaplan AP. Factor XII-independent activation of the bradykininforming cascade: Implications for the pathogenesis of hereditary angioedema types I and II. J Allergy Clin Immunol. (2013) 132:470–5. 10.1016/j.jaci.2013.03.026 23672780

[76] Nussberger J Cugno M Amstutz C Cicardi M Pellacani A Agostoni A. Plasma bradykinin in angio-oedema. Lancet. (1998) 351:1693–7. 10.1016/S0140-6736(97)09137-X 9734886

[77] Nussberger J Cugno M Cicardi M Agostoni A. Local bradykinin generation in hereditary angioedema. J Allergy Clin Immunol. (1999) 104:1321–2. 10.1016/S0091-6749(99)70030-8 10589018

[78] Cugno M Zanichelli A Foieni F Caccia S Cicardi M. C1-inhibitor deficiency and angioedema: Molecular mechanisms and clinical progress. Trends Mol Med. (2009) 15:69–78. 10.1016/j.molmed.2008.12.001 19162547

[79] Riedl MA. Hereditary angioedema with normal C1-INH (HAE type III). J Allergy Clin Immunol Pract. (2013) 1:427–32. 10.1016/j.jaip.2013.06.004 24565612

[80] Cichon S Martin L Hennies HC Müller F Van Driessche K Karpushova A. Increased activity of coagulation factor XII (Hageman factor) causes hereditary angioedema type III. Am J Hum Genet. (2006) 79:1098–104. 10.1086/509899 17186468 PMC1698720

[81] Cugno M Castelli R Cicardi M. Angioedema due to acquired C1-inhibitor deficiency: a bridging condition between autoimmunity and lymphoproliferation. Autoimmun Rev. (2008) 8:156–9. 10.1016/j.autrev.2008.05.003 19014872

[82] Wu MA Castelli R. The Janus faces of acquired angioedema: C1-inhibitor deficiency, lymphoproliferation and autoimmunity. Clin Chem Lab Med. (2016) 54:207–14. 10.1515/cclm-2015-0195 26068904

[83] Liau Y Chua I Kennedy MA Maggo S. Pharmacogenetics of angiotensin converting enzyme inhibitor - induced angioedema. Clin Exp Allergy. (2019) 49:142–54. 10.1111/cea.13326 30537422

[84] Nussberger J Cugno M Cicardi M. Bradykinin-mediated angioedema. N Engl J Med. (2002) 347:621–2. 10.1056/NEJM200208223470820 12192030

[85] Sala-Cunill A Björkqvist J Senter R Guilarte M Cardona V Labrador M. Plasma contact system activation drives anaphylaxis in severe mast cell-mediated allergic reactions. J Allergy Clin Immunol. (2014) 135:1031–43. 10.1016/j.jaci.2014.07.057 25240785

[86] Davis AEIII. C1 inhibitor and hereditary angioneurotic edema. Annu Rev Immunol. (1988) 6:595–628. 10.1146/annurev.iy.06.040188.003115 3289579

[87] Cugno M Bergamaschini L Uziel L Cicardi M Agostoni A Jie AF. Haemostasis contact system and fibrinolysis in hereditary angioedema (C1-inhibitor deficiency). J Clin Chem Clin Biochem. (1988) 26:423–7. 10.1515/cclm.1988.26.7.423 3146615

[88] Cugno M Cicardi M Coppola R Agostoni A. Activation of factor XII and cleavage of high molecular weight kininogen during acute attacks in hereditary and acquired C1-inhibitor deficiencies. Immunopharmacology. (1996) 33:361–4. 10.1016/0162-3109(96)00086-0 8856187

[89] Cugno M Nuijens J Hack E Eerenberg A Frangi D Agostoni A. Plasma levels of C1-inhibitor complexes and cleaved C1-inhibitor in patients with hereditary angioneurotic edema. J Clin Invest. (1990) 85:1215–20. 10.1172/JCI114555 2318974 PMC296554

[90] Cugno M Cicardi M Bottasso B Coppola R Paonessa R Mannucci PM. Activation of the coagulation cascade in C1-inhibitor deficiencies. Blood. (1997) 89:3213–8. 9129025

[91] Cugno M Zanichelli A Bellatorre AG Griffini S Cicardi M. Plasma biomarkers of acute attacks in patients with angioedema due to C1-inhibitor deficiency. Allergy. (2009) 64:254–7. 10.1111/j.1398-9995.2008.01859.x 19076541

[92] Cugno M Cicardi M Agostoni A. Activation of the contact system and fibrinolysis in autoimmune acquired angioedema: a rationale for prophylactic use of tranexamic acid. J Allergy Clin Immunol. (1994) 93:870–6. 10.1016/0091-6749(94)90380-8 8182230

[93] Cugno M Hack CE de Boer JP Eerenberg AJ Agostoni A Cicardi M. Generation of plasmin during acute attacks of hereditary angioedema. J Lab Clin Med. (1993) 121:38–43. 8426080

[94] Ehringer WD Edwards MJ Miller FN Mechanisms of a-thrombin, histamine, and bradykinin induced endothelial permeability. J Cell Physiol. (1996) 167:562 10.1002/(SICI)1097-4652(199606)167:3&lt;562::AID-JCP20&gt;3.0.CO;2-4 8655610

[95] Donaldson VH. Blood coagulation and related plasma enzymes in inflammation. Ser Haemat. (1970) 3:39. 4943386

[96] De Maat S Hofman ZLM Maas C Hereditary angioedema: the plasma contact system out of control. J Thromb Haemost. (2018) 16:1674–85. 10.1111/jth.14209 29920929

[97] Maas C Renné T. Coagulation factor XII in thrombosis and inflammation. Blood. (2018) 131:1903–9. 10.1182/blood-2017-04-569111 29483100

[98] Varga L Farkas H. Comprehensive study into the activation of the plasma enzyme systems during attacks of hereditary angioedema due to C1-inhibitor deficiency. Orphanet J Rare Dis. (2015) 10:132. 10.1186/s13023-015-0351-5 26452350 PMC4600308

[99] Reshef A Zanichelli A Longhurst H Relan A Hack CE Elevated D-dimers in attacks of hereditary angioedema are not associated with increased thrombotic risk. Allergy. (2015) 70:506–13. 10.1111/all.12587 25640891 PMC4409094

[100] Mansi M Zanichelli A Coerezza A Suffritti C Wu MA Vacchini R. Presentation, diagnosis and treatment of angioedema without wheals: a retrospective analysis of a cohort of 1058 patients. J Intern Med. (2015) 277:585–93. 10.1111/joim.12304 25196353

[101] Kridin K Shihade W Bergman R. Mortality in patients with bullous pemphigoid: a retrospective cohort study, systematic review and meta-analysis. Acta Derm Venereol. (2018) 99:72–7. 10.2340/00015555-2930 29963683

[102] Rozenblat M Halaj A Rozenblat T Fisher S Sah M Dodiuk-Gad RP. Mortality and risk factors among Israeli bullous pemphigoid patients. Arch Dermatol Res. (2019) 311:19–27. 10.1007/s00403-018-1875-z 30382340

[103] Roujeau JC Lok C Bastuji-Garin S Mhalla S Enginger V Bernard P. High risk of death in elderly patients with extensive bullous pemphigoid. Arch Dermatol. (1998) 134:465–9. 10.1001/archderm.134.4.465 9554299

[104] Bernard P Antonicelli F. Bullous Pemphigoid: A Review of its Diagnosis, Associations and Treatment. Am J Clin Dermatol. (2017) 18:513–28. 10.1007/s40257-017-0264-2 28247089

[105] Kridin K Ludwig RJ. The growing incidence of bullous pemphigoid: overview and potential explanations. Front Med. (2018) 5:220. 10.3389/fmed.2018.00220 30177969 PMC6109638

[106] della Torre R Combescure C Cortes B Marazza G Beltraminelli H Naldi L. Clinical presentation and diagnostic delay in bullous pemphigoid: a prospective nationwide cohort. Br J Dermatol. (2012) 167:1111–7. 10.1111/j.1365-2133.2012.11108.x 22709136

[107] Yancey KB Egan CA. Pemphigoid: clinical, histologic, immunopathologic, and therapeutic considerations. JAMA. (2000) 284:350–6. 10.1001/jama.284.3.350 10891967

[108] Bernard P Reguiai Z Tancrède-Bohin E Cordel N Plantin P Pauwels C. Risk factors for relapse in patients with bullous pemphigoid in clinical remission: a multicenter, prospective, cohort study. Arch Dermatol. (2009) 145:537–42. 10.1001/archdermatol.2009.53 19451497

[109] Yancey KB. The pathophysiology of autoimmune blistering diseases. J Clin Invest. (2005) 115:825–8. 10.1172/JCI200524855 15841169 PMC1070439

[110] Bagci IS Horváth ON Ruzicka T Sárdy M. Bullous pemphigoid. Autoimmun Rev. (2017) 16:445–55. 10.1016/j.autrev.2017.03.010 28286109

[111] Hertl M Eming R Veldman C. T cell control in autoimmune bullous skin disorders. J Clin Invest. (2006) 116:1159–66. 10.1172/JCI28547 16670756 PMC1451217

[112] Lo Schiavo A Ruocco E Brancaccio G Caccavale S Ruocco V Wolf R. Bullous pemphigoid: etiology, pathogenesis, and inducing factors: facts and controversies. Clin Dermatol. (2013) 31:391–9. 10.1016/j.clindermatol.2013.01.006 23806156

[113] Le Jan S Plée J Vallerand D Dupont A Delanez E Durlach A. Innate immune cell-produced IL-17 sustains inflammation in bullous pemphigoid. J Invest Dermatol. (2014) 134:2908–17. 10.1038/jid.2014.263 24945093 PMC4227922

[114] Arakawa M Dainichi T Ishii N Hamada T Karashima T Nakama T. Lesional Th17 cells and regulatory T cells in bullous pemphigoid. Exp Dermatol. (2011) 20:1022–24. 10.1111/j.1600-0625.2011.01378.x 22017210

[115] Chakievska L Holtsche MM Künstner A Goletz S Petersen BS Thaci D. IL-17A is functionally relevant and a potential therapeutic target in bullous pemphigoid. J Autoimmun. (2019) 96:104–12. 10.1016/j.jaut.2018.09.003 30219389

[116] Gambichler T Tsitlakidon A Skrygan M Höxtermann S Susok L Hessam S. T regulatory cells and other lymphocyte subsets in patients with bullous pemphigoid. Clin Exp Dermatol. (2017) 42:632–7. 10.1111/ced.13135 28590036

[117] Liu Z Dang E Li B Qiao H Jin L Zhang J. Dysfunction of CD19+CD24hiCD27+ B regulatory cells in patients with bullous pemphigoid. Sci Rep. (2018) 8:703. 10.1038/s41598-018-19226-z 29335495 PMC5768798

[118] Chiorean RM Baican A Mustafa MB Lischka A Leucuta DC Feldrihan V. Complement-activating capacity of autoantibodies correlates with disease activity in bullous pemphigoid patients. Front Immunol. (2018) 9:2687. 10.3389/fimmu.2018.02687 30524436 PMC6257046

[119] Karsten CM Beckmann T Holtsche MM Tillmann J Tofern S Schulze FS. Tissue destruction in bullous pemphigoid can be complement independent and may be mitigated by C5aR2. Front Immunol. (2018) 9:488. 10.3389/fimmu.2018.00488 29599777 PMC5862877

[120] Marzano AV Tedeschi A Fanoni D Bonanni E Venegoni L Berti E. Activation of blood coagulation in bullous pemphigoid: role of eosinophils, and local and systemic implications. Br J Dermatol. (2009) 160:266–72. 10.1111/j.1365-2133.2008.08880.x 18945300

[121] Marzano AV Tedeschi A Spinelli D Fanoni D Crosti C Cugno M. Coagulation activation in autoimmune bullous diseases. Clin Exp Immunol. (2009) 158:31–6. 10.1111/j.1365-2249.2009.03989.x 19737228 PMC2759056

[122] Cugno M Tedeschi A Crosti C Marzano AV. Activation of blood coagulation in autoimmune skin disorders. Exp Rev Clin Immunol. (2009) 5:605–13. 10.1586/eci.09.40 20477646

[123] Marzano AV Tedeschi A Berti E Crosti C Cugno M. Activation of coagulation in bullous pemphigoid and other eosinophil-related inflammatory skin diseases. Clin Exp Immunol. (2011) 165:44–50. 10.1111/j.1365-2249.2011.04391.x 21488867 PMC3110320

[124] de Graauw E Sitaru C Horn M Borradori L Yousefi S Simon HU. Evidence for a role of eosinophils in blister formation in bullous pemphigoid. Allergy. (2017) 72:1105–13. 10.1111/all.13131 28135772

[125] Kridin K Peripheral eosinophilia in bullous pemphigoid: prevalence and influence on the clinical manifestation. Br J Dermatol. (2018) 79:1141–7. 10.1111/bjd.16679 29663327

[126] Giusti D Gatouillat G Le Jan S Plee J Bernard P Antonicelli F. Eosinophil Cationic Protein (ECP), a predictive marker of bullous pemphigoid severity and outcome. Sci Rep. (2017) 7:4833. 10.1038/s41598-017-04687-5 28684769 PMC5500584

[127] Tedeschi A Marzano AV Lorini M Balice Y Cugno M. Eosinophil cationic protein levels parallel coagulation activation in the blister fluid of patients with bullous pemphigoid. J Eur Acad Dermatol Venereol. (2015) 29:813–7. 10.1111/jdv.12464 24650303

[128] Wakugawa M Nakamura K Hino H Toyama K Hattori N Okochi H. Elevated levels of eotaxin and interleukin-5 in blister fluid of bullous pemphigoid: correlation with tissue eosinophilia. Br J Dermatol. (2000) 143:112–6. 10.1046/j.1365-2133.2000.03599.x 10886144

[129] Engineer L Bhol K Kumari S Razzaque Ahmed A. Bullous pemphigoid: interaction of interleukin 5, anti-basement membrane zone antibodies and eosinophils. A preliminary observation. Cytokine. (2001) 13:32–8. 10.1006/cyto.2000.0791 11145840

[130] Frezzolini A Cianchini G Ruffelli M Cadoni S Puddu P De Pità O. Interleukin-16 expression and release in bullous pemphigoid. Clin Exp Immunol. (2004) 137:595–600. 10.1111/j.1365-2249.2004.02570.x 15320912 PMC1809150

[131] Amber KT Valdebran M Kridin K Grando SA. The role of eosinophils in bullous pemphigoid: a developing model of eosinophil pathogenicity in mucocutaneous disease. Front Med. (2018) 5:201. 10.3389/fmed.2018.00201 30042946 PMC6048777

[132] Shimanovich I Mihai S Oostingh GJ Ilenchuk TT Bröcker EB Opdenakker G. Granulocyte-derived elastase and gelatinase B are required for dermal-epidermal separation induced by autoantibodies from patients with epidermolysis bullosa acquisita and bullous pemphigoid. J Pathol. (2004) 204:519–27. 10.1002/path.1674 15538734

[133] Ståhle-Bäckdahl M Inoue M Guidice GJ Parks WC. 92-kD gelatinase is produced by eosinophils at the site of blister formation in bullous pemphigoid and cleaves the extracellular domain of recombinant 180-kD bullous pemphigoid autoantigen. J Clin Invest. (1994) 93:2022–30. 10.1172/JCI117196 8182134 PMC294314

[134] Borrego L Maynard B Peterson EA George T Iglesias L Peters MS. Deposition of eosinophil granule proteins precedes blister formation in bullous pemphigoid. Comparison with neutrophil and mast cell granule proteins. Am J Pathol. (1996) 148:897–909. 8774144 PMC1861728

[135] Davis MD Plager DA George TJ Weiss EA Gleich GJ Leiferman KM. Interactions of eosinophil granule proteins with skin: limits of detection, persistence, and vasopermeabilization. J Allergy Clin Immunol. (2003) 112:988–94. 10.1016/j.jaci.2003.08.028 14610493

[136] Lin L Hwang BJ Culton DA Li N Burette S Koller BH. Eosinophils mediate tissue injury in autoimmune skin disease bullous pemphigoid. J Invest Dermatol. (2017) 138:1032–43. 10.1016/j.jid.2017.11.031 29246800 PMC7531612

[137] Cozzani E Gasparini G Di Zenzo G Parodi A. Immunoglobulin E and bullous pemphigoid. Eur J Dermatol. (2018) 28:440–8. 10.1684/ejd.2018.3366 30325326

[138] Messingham KN Holahan HM Frydman AS Fullenkamp C Srikantha R Fairley JA. Human eosinophils express the high affinity IgE receptor, FcεRI, in bullous pemphigoid. PLoS ONE. (2014) 9:e107725. 10.1371/journal.pone.0107725 25255430 PMC4177878

[139] James T Salman S Stevenson B Bundell C Kelly G Nolan D. IgE blockade in autoimmunity: omalizumab induced remission of bullous pemphigoid. Clin Immunol. (2018) 198:54–6. 10.1016/j.clim.2018.12.015 30557620

[140] Kremer N Snast I Cohen ES Hodak E Mimouni D Lapidoth M Rituximab and omalizumab for the treatment of bullous pemphigoid: a systematic review of the literature. Am J Clin Dermatol. (2018) 2018:6 10.1007/s40257-018-0401-6 30421306

[141] Rudrich U Gehring M Papakonstantinou E Rabenhorst A Engmann J Kapp A. Eosinophils are a major source of interleukin-31 in bullous pemphigoid. Acta Derm Venereol. (2018) 98:766–71. 10.2340/00015555-2951 29693698

[142] Kunsleben N Rudrich U Gehring M Novak N Kapp A Raap U. IL-31 induces chemotaxis, calciummobilization, release of reactive oxygen species, and CCL26 in eosinophils, which are capable to release IL-31. J Invest Dermatol. (2015) 135:1908–11. 10.1038/jid.2015.106 25789701

[143] Bonciani D Quintarelli L Del Bianco E Bianchi B Caproni M. Serum levels and tissue expression of interleukin-31 in dermatitis herpetiformis and bullous pemphigoid. J Dermatol Sci. (2017) 87:210–2. 10.1016/j.jdermsci.2017.04.008 28465041

[144] Salz M Haeberle S Hoffmann J Enk AH Hadaschik EN. Elevated IL-31 serum levels in bullous pemphigoid patients correlate with eosinophil numbers and are associated with BP180-IgE. J Dermatol Sci. (2017) 87:309–11. 10.1016/j.jdermsci.2017.07.019 28823642

[145] Zebrowska A Wagrowska-Danilewicz M Danilewicz M Wieczfinska J Pniewska E Zebrowski M. Tissue factor in dermatitis herpetiformis and bullous pemphigoid: link between immune and coagulation system in subepidermal autoimmune bullous diseases. Mediat Inflamm. (2015) 2015:870428. 10.1155/2015/870428 27057091 PMC4709721

[146] Narbutt J Waszczykowska E Lukamowicz J Sysa-Jedrzejowska A Kobos J Zebrowska A. Disturbances of the expression of metalloproteinases and their tissue inhibitors cause destruction of the basement membrane in pemphigoid. Pol J Pathol. (2006) 57:71–6. 17019968

[147] So AK Varisco PA Kemkes-Matthes B Herkenne-Morard C Chobaz-Péclat V Gerster JC. Arthritis is linked to local and systemic activation of coagulation and fibrinolysis pathways. J Thromb Haemost. (2003) 1:2510–5. 10.1111/j.1538-7836.2003.00462.x 14675085

[148] Kume K Yamasaki M Tashiro M Yoshikawa I Otsuki M. Activations of coagulation and fibrinolysis secondary to bowel inflammation in patients with ulcerative colitis. Intern Med. (2007) 46:1323–9. 10.2169/internalmedicine.46.0237 17827828

[149] Liaw PC Esmon CT Kahnamoui K Schmidt S Kahnamoui S Ferrell G. Patients with severe sepsis vary markedly in their ability to generate activated protein C. Blood. (2004) 104:3958–64. 10.1182/blood-2004-03-1203 15319291

[150] Garcia JG Siflinger-Birnboim A Bizios R Del Vecchio PJ Fenton JW II Malik AB. Thrombin-induced increase in albumin permeability across the endothelium. J Cell Physiol. (1986) 128:96–104. 10.1002/jcp.1041280115 3722274

[151] DeMichele MA Moon DG Fenton JW II Minnear FL. Thrombin's enzymatic activity increases permeability of endothelial cell monolayers. J Appl Physiol. (1990) 69:1599–606. 10.1152/jappl.1990.69.5.1599 2272952

[152] Saibeni S Ciscato C Vecchi M Boscolo Anzoletti M Kaczmarek E Caccia S. Antibodies to tissue-type plasminogen activator (t-PA) in patients with inflammatory bowel disease: high prevalence, interactions with functional domains of t-PA and possible implications in thrombosis. J Thromb Haemost. (2006) 4:1510–6. 10.1111/j.1538-7836.2006.01970.x 16839347

[153] Ingegnoli F Fantini F Griffini S Soldi A Meroni PL Cugno M. Anti-tumor necrosis factor alpha therapy normalizes fibrinolysis impairment in patients with active rheumatoid arthritis. Clin Exp Rheumatol. (2010) 28:254–7. 20483049

[154] Marzano AV Tedeschi A Polloni I Crosti C Cugno M. Prothrombotic state and impaired fibrinolysis in bullous pemphigoid, the most frequent autoimmune blistering disease. Clin Exp Immunol. (2013) 171:76–81. 10.1111/j.1365-2249.2012.04674.x 23199326 PMC3530098

[155] Yang YW Chen YH Xirasagar S Lin HC. Increased risk of stroke in patients with bullous pemphigoid: a population-based follow-up study. Stroke. (2011) 42:319–23. 10.1161/STROKEAHA.110.596361 21164122

[156] Joly P Benichou J Lok C Hellot MF Saiag P Tancrede-Bohin E. Prediction of survival for patients with bullous pemphigoid: a prospective study. Arch Dermatol. (2005) 141:691–8. 10.1001/archderm.141.6.691 15967914

[157] Rosendaal FR Van Hylckama Vlieg A Doggen CJM. Venous thrombosis in the elderly. J Thromb Haemost. (2007) 5:310–7. 10.1111/j.1538-7836.2007.02489.x 17635742

[158] Langan SM Hubbard R Fleming K West J. A population-based study of acute medical conditions associated with bullous pemphigoid. Br J Dermatol. (2009) 161:1149–52. 10.1111/j.1365-2133.2009.09350.x 19681857

[159] Cugno M Marzano AV Bucciarelli P Balice Y Cianchini G Quaglino P. Increased risk of venous thromboembolism in patients with bullous pemphigoid. The INVENTEP (INcidence of VENous ThromboEmbolism in bullous Pemphigoid) study. Thromb Haemost. (2016) 115:193–9. 10.1160/TH15-04-0309 26245987

[160] Kibsgaard L Bay B Deleuran M Vestergaard C. A retrospective consecutive case-series study on the effect of systemic treatment, length of admission time, and co-morbidities in 98 bullous pemphigoid patients admitted to a tertiary centre. Acta Derm Venereol. (2015) 95:307–11. 10.2340/00015555-1925 24979241

